# Insights from quantitative and mathematical modelling on the proposed WHO 2030 goals for Chagas disease

**DOI:** 10.12688/gatesopenres.13069.1

**Published:** 2019-09-17

**Authors:** Collaborating Group on Chagas Disease Modelling

**Keywords:** Chagas disease, WHO guidelines, Elimination as a public health problem, intradomiciliary transmission interruption, trypanocidal treatment, NTD Modelling Consortium

## Abstract

Chagas disease (CD) persists as one of the neglected tropical diseases (NTDs) with a particularly large impact in the Americas. The World Health Organization (WHO) recently proposed goals for CD elimination as a public health problem to be reached by 2030 by means of achieving intradomiciliary transmission interruption (IDTI), blood transfusion and transplant transmission interruption, diagnostic and treatment scaling-up and prevention and control of congenital transmission. The NTD Modelling Consortium has developed mathematical models to study *Trypanosoma cruzi *transmission dynamics and the potential impact of control measures. Modelling insights have shown that IDTI is feasible in areas with sustained vector control programmes and no presence of native triatomine vector populations. However, IDTI in areas with native vectors it is not feasible in a sustainable manner. Combining vector control with trypanocidal treatment can reduce the timeframes necessary to reach operational thresholds for IDTI (<2% seroprevalence in children aged <5 years), but the most informative age groups for serological monitoring are yet to be identified. Measuring progress towards the 2030 goals will require availability of vector surveillance and seroprevalence data at a fine scale, and a more active surveillance system, as well as a better understanding of the risks of vector re-colonization and disease resurgence after vector control cessation. Also, achieving scaling-up in terms of access to treatment to the expected levels (75%) will require a substantial increase in screening asymptomatic populations, which is anticipated to become very costly as CD prevalence decreases. Further modelling work includes refining and extending mathematical models (including transmission dynamics and statistical frameworks) to predict transmission at a sub-national scale, and developing quantitative tools to inform IDTI certification, post-certification and re-certification protocols. Potential perverse incentives associated with operational thresholds are discussed. These modelling insights aim to inform discussions on the goals and treatment guidelines for CD.

## Abbreviations

BNZ, benznidazole; BTT, blood transfusion transmission; CD, Chagas disease; CTI, congenital transmission interruption; EMTCT Plus, elimination of mother-to-child transmission of HIV, syphilis, Chagas, and perinatal hepatitis B; EPHP, elimination as a public health problem; FOI, force-of-Infection; IDTI, intradomiciliary transmission interruption; IRS, indoor residual spraying; NFX, nifurtimox; NTD, neglected tropical disease; PAHO, Pan American Health Organization; PCR, polymerase chain reaction; PPC, proportion of parasitological cure; qPCR, quantitative PCR; R&D, research and development; TTT, tissue transplant transmission; WHO, World Health Organization; WISCC, World Information System for the Control of Chagas Disease; 95% CI, ninety-five percent confidence interval.

## Disclaimer

The views and opinions expressed in this article are those of the authors and do not necessarily reflect those of the World Health Organization. Publication in Gates Open Research does not imply endorsement by the Gates Foundation.

## Background

With an estimated 8–10 million cases worldwide, Chagas disease (CD; also known as American trypanosomiasis) remains a major cause of heart disease morbidity, mortality and economic burden, particularly in endemic Latin American countries ^1^. CD is a parasitic disease caused by the protozoan *Trypanosoma cruzi*, and transmitted mainly by domiciliated triatomine (Reduviidae) vectors (kissing bugs) in tropical areas of the Americas. However, along with the urbanization process in recent decades, other transmission routes, such as blood transfusion, organ transplant and congenital have become important in both endemic and non-endemic countries ^2^. The disease is characterized by a long asymptomatic period (years to decades) before reaching the full set of clinical manifestations that include heart failure, arrythmias, stroke, digestive complications and other conditions that increase the risk of death ^3^.

From the beginning of the Southern Cone Initiative in the 1990s, most endemic countries have made great progress towards the control of *T. cruzi* transmission by implementing mainly vector control (chiefly through indoor residual-insecticide spraying (IRS)) and blood transfusion control via donor screening. So far, 11 out of the 21 recognised endemic countries have been certified as having reached intradomiciliary transmission interruption (IDTI) ^4^. However, the real impact of such interventions has not been rigorously documented and quantified, and various concerns have been raised around the relationship between reaching the various operational thresholds that have been proposed and truly achieving interruption/elimination of transmission and reduction in morbidity ^5^. Also, the increased health-care demands from the chronically-affected populations, and the limited offer of diagnosis and trypanocidal and supportive treatment pose additional challenges to CD control.

The World Health Organization (WHO) has set goals for the control of CD by 2030 in both endemic and non-endemic countries, including achieving the target of elimination as a public health problem (EPHP), the interruption of the various transmission routes and the scale-up of diagnosis and treatment strategies (a summary of these goals is presented in Table 1). In order to help evaluate progress towards and feasibility of these goals, mathematical modellers from different countries have joined forces, under the invitation of the Bill & Melinda Gates Foundation-funded NTD Modelling Consortium, to contribute to a joint analysis of mathematical modelling insights to support the CD WHO goals for 2030. In February 2018 a workshop on “How can modelling contribute to achieving the goals for Chagas disease in the Horizon 2020 and beyond?” was held at Imperial College London, with participants contributing ideas on how to quantitatively inform global progress on control and elimination of CD. In this document, we consolidate the main points from these discussions, involving ecological and epidemiological modellers and researchers from Imperial College London and Sussex University UK, Princeton University and University of Pennsylvania USA, University of Perpignan Via Domitia France, University of La Plata and CONICET Argentina, and Fundação Oswaldo Cruz – Fiocruz, Brazil.

**Table 1.** Summary of modelling insights and challenges for reaching the WHO 2030 goals for Chagas disease (CD).

Current WHO Goal (2020)	1) Interruption of intradomiciliary (vectorial) transmission in the Americas 2) Interruption of transfusional transmission by blood/blood products in the Americas, Europe and Western Pacific
WHO new targets (2030)	1) Elimination as a public health problem (EPHP, 20 endemic countries + 75% access to treatment); 2) Interruption of intradomiciliary (vectorial) transmission (IDTI) with 0% colonization of dwellings and 0% incidence of *T. cruzi*-infected persons; 3) Elimination of blood transfusion-related transmission (BTT) in 100% of the target countries; 4) Elimination of tissue transplant-related transmission (TTT) (in 100% of the target countries; 5) Interruption of congenital transmission (CTI) of Chagas disease: 90% screening coverage and treatment to women, and when infected, screening of their new-borns and siblings.
Are the new targets technically feasible under the current intervention strategy?	Achieving IDTI in areas with coexistent transmission by native vectors is not feasible in a sustainable manner; BTT and TTT are potentially achievable but a clear protocol of strategies for achieving these goals is needed; 100% treatment of women of childbearing age is not feasible (a proportion of women have contraindications and abandonment of treatment has been estimated at 20%). 100% diagnosis in new-borns is not feasible (parasitological test sensitivity <30% and infrastructure for more sensitive tests (qPCR) is far from optimal).
If not, what is required to achieve the targets?	When current vector control strategy is combined with annual trypanocidal treatment in 10% of the infected human population, the seroprevalence (operational) criterion (<2% in under 5-year old children) could be achieved in 1/3 of the time (11 years in highly-endemic settings). In order to achieve high treatment coverage, various parameters interact (probability of screening, sensitivity of the test, proportion of people receiving and adhering to complete treatment course and drug efficacy). The lowest of all these is currently screening (access to diagnosis <1%).
Are current tools able to reliably measure the targets?	Force-of-Infection (FOI) models using age-stratified seroprevalence data are a promising tool to measure efficacy of IDTI. Tools are required to evaluate BTT, TTT and CTI. The “0% colonization” target can only be measured very imperfectly with current vector-surveillance tools.
What are the biggest unknowns?	Prevalence of infection and house infestation in many areas; role of native vector species in transmission; resistance status of vector populations to currently available insecticides; disentangling the impact of chemical vector control from that of housing improvement; efficacy of trypanocidal treatment under various rates of abandonment.
What are the biggest risks?	Slacking elimination efforts before reaching EPHP; spread of insecticide resistance; not being able to identify and treat congenital cases; treatment of children with false positive results.

## Insights gained from quantitative and mathematical modelling analyses

### Intra-domiciliary transmission interruption (IDTI)

A main planned target is achieving IDTI in endemic countries with 0% colonization of dwellings and 0% incidence of *T. cruzi*-infected persons ( Table 1). The accumulated body of knowledge from the modelling work that has been undertaken by these groups over the last years tends to agree on various aspects, particularly the important progress made towards IDTI. Reductions of incidence and disease burden through the control or elimination of introduced/non-native *Triatoma infestans* (in some areas of the Southern Cone such as Brazil, Uruguay, and pockets of Paraguay, Chile, Peru, and Argentina) and of *Rhodnius prolixus* (in Central America and some pockets in Northern South America) have been achieved by a combination of IRS, locally practicable environmental management strategies and housing improvement, as initially suggested by pioneer mathematical modelling ^6^. However, enormous challenges and limitations persist in terms of sustainability, data availability to monitor progress or re-emergence (which includes vector re-introduction (or resurgence from residual foci) and re-emergence of transmission), and clarity in the specific strategies to be undertaken towards the achievement of the 2030 goals.

IDTI is potentially achievable in epidemiological settings with exclusively domiciliary (i.e., non-native) vectors and no insecticide resistance. Pioneer work also illustrated an application of triatomine-population modelling to optimize IRS for vector control, suggesting how to determine the optimal timing of spraying for the control of *T. infestans* in Argentina, depending on the season and the structure of the triatomine population ^7^. Using a similar approach with current techniques will be useful to help programmes to better target IRS strategies against domiciliated vectors. The critical (threshold) number of triatomines per house related to transmission risk, and how this can be used to prioritize vector control campaigns, has been investigated. A relationship between house infestation (proportion of houses infested) and the number of triatomines/house was fitted to data from various locations prior to vector control and applied to cases where there was only one triatomine species present in the dwellings, as well as to situations with mixtures of species and developmental stages, various types of houses and bug densities per house. These relationships can be improved when data are stratified (if available) with some critical co-variates, such as house-construction materials, number of humans and zoonotic hosts in the dwellings, and IRS status ^8, 9^. Understanding further these relationships taking into account other variables (such as the distribution of triatomines per house, the triatomine species, the time elapsed after intervention, etc.), will be crucial to determine operational thresholds that lead to cost optimisation ^10^.

However, large areas of endemic countries with domiciliated vector species have sylvatic populations (for which traditional vector control measures are less effective). An example is *Rhodnius prolixus,* a vector that has been targeted for elimination in Central and South America. Unlike Central America, where *R. prolixus* is strictly domiciliated ^11^, large areas of Colombia and Venezuela have *R. prolixus* as the most prominent sylvatic species. In these areas re-colonisation occurs readily between 1 to 67 months after IRS ^12^. In the presence of sylvatic populations, there is a continuous introduction and colonisation of domiciliary and peri-domiciliary habitats; in these areas, traditional vector control is not feasible in a sustainable manner. Additionally, even if the domiciliated vector species are eliminated, their niche could be taken over by other sylvatic vector species.

Various studies on routine vector surveillance have demonstrated that the currently used methods have low sensitivity and greatly underestimate vector density, infestation and infection rates; vector surveillance may be capturing half of infestations – and, most likely, most bugs within a house ^13, 14^. These shortcomings may have greater impact in low-prevalence and post-intervention settings.

**Figure 1.**
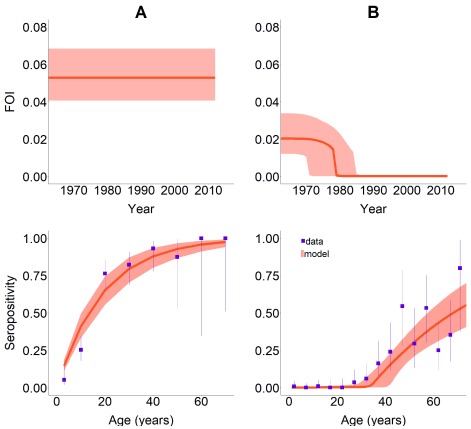
Force-of-Infection (FOI) models fitted to seroprevalence data. Upper panels represent the (modelled) historical FOI. Lower panels present the data (solid squares) and the modelled seroprevalence (orange shaded area) in: ( **A**) a non-intervened area with a long-established endemic situation and ( **B**) a successfully intervened area. This figure has been reproduced from 15 under a Creative Common Attribution 4.0 International Licence (CC BY 4.0).

Measuring 0% incidence requires analysis of seroprevalence studies, but diagnostic tests do not have perfect sensitivity and specificity. Investment in Research and Development (R&D) is essential to improve the performance of serology-based tests, particularly in near-elimination (low-prevalence) settings. Also, hierarchical models can be used to estimate test performance parameters (sensitivity and specificity) and then correct infection frequency ^14^. The most informative age classes for seromonitoring should be identified, and strategies developed for monitoring the long-term response to control. Modelling the historical force-of-infection (FOI; the per susceptible incidence rate) using population representative seroprevalence studies, is a promising quantitative tool to measure trends in incidence and achievement of operational thresholds for transmission interruption as done in Peru and Colombia ^15, 16^ (e.g. <2% seroprevalence in under 5-year-olds) ( Figure 1). However, even if IDTI were achieved, the likely presence of remaining vector populations, the protracted temporal scale of *T. cruzi* transmission (decades), and the long asymptomatic period of infection, can lead to many years passing before parasite re-emergence is noticed. FOI (catalytic) models are also a promising tool to estimate time to resurgence when a strategy has not been sufficiently effective, as applied in La Joya, Peru ^15^ and the Bolivian Chaco ^17^.

**Figure 2.**
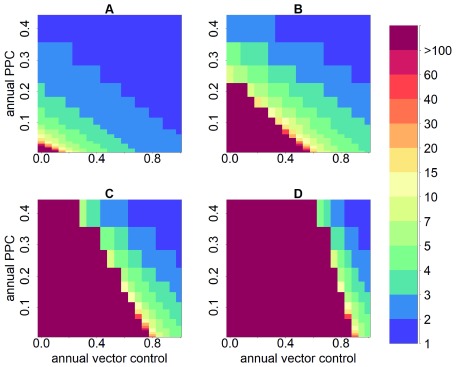
Combined impact of vector control and effective parasite clearance. Annual vector control defines the proportion by which vector density is annually reduced (0–100%); parasite clearance is measured as proportion of parasitological cure (PPC); annual PPC defines the proportion of humans effectively treated annually with trypanocidal treatment, i.e., the percentage of the infected human population achieving parasitological cure (0–40%). The impact of the combined strategies is measured on the number of years necessary to reduce seroprevalence in children aged <5 years to <2% (the operational serological criterion for intradomiciliary transmission interruption), as represented by the colour scale. The panels represent: ( **A**) low; ( **B**) moderate; ( **C**) high; and ( **D**) very high endemicity levels. This figure has been reproduced from 17 under a Creative Common Attribution 4.0 International Licence (CC BY 4.0).

Modelling studies have indicated that potentially combining highly effective vector control with trypanocidal treatment of humans residing in endemic areas would substantially reduce the time required to achieve operational serological thresholds for IDTI as well as infection incidence and prevalence ^18^ ( Figure 2). Understanding the implications of this combination of interventions for achieving elimination of transmission and EPHP needs further work.

### Understanding the role of sylvatic vectors

Native triatomines maintain extensive enzootic *T. cruzi* transmission cycles from the United States of America to Patagonia, including vast areas where the three main domestic vectors of human CD ( *Triatoma infestans, T. dimidiata,* and *Rhodnius prolixus*) occur in the wild, with the potential for invading, infesting, and re-infesting dwellings. Challenges remain concerning the role and magnitude of rural and urban transmission, which are harder to quantify. In vast areas of endemic countries, intradomicilary transmission is due to ‘sylvatic’ species that do not colonize domiciles but only make occasional visits (also called ‘intrusive’ species) ^19^. This has led to researchers to suggest an alternative classification of triatomines that captures their complexity but is still operationally relevant for surveillance ^20^.

Recent studies have modelled the relative roles of some candidate variables on house invasion by sylvatic triatomines ^21^. Modelling studies suggest that a better comprehension of vectorial transmission in rural and urban settings would require understanding and quantifying of two different forms of vector dispersal, namely, dispersal between sylvatic and non-sylvatic habitats and diffusive dispersal within cities ^22^.

A promising avenue is the application of orthogonal polynomials methods to predict triatomine dispersal based upon exclusively life-history data of each triatomine species ^23^. Tackling transmission by native vectors may necessitate alternative control strategies, which would require measuring dispersal and evaluating the efficacy of strategies such as those used for other vector-borne diseases, e.g. (impregnated) nets. Examples of the application of transmission dynamics and statistical models to evaluate both dispersal and the potential efficacy of such alternative strategies in comparison to typical IRS have been discussed ^22^.

### Scaling up screening and treatment strategies

Another main target planned is achieving 75% access to trypanocidal treatment in *T. cruzi-*infected people with medical indications ( Table 1). Trypanocidal treatment with benznidazole (BNZ) or nifurtimox (NFX) has been aimed at both reducing parasitaemia and curbing disease progression. So far, there is limited evidence on the efficacy of drugs for these.

Monotherapy with BNZ has been proven to reduce parasitaemia in up to 86.7% of treated patients ^24^. However, it is known that the trypanocidal effect of BNZ varies across regions ^25, 26^. The evidence of trypanocidal efficacy for NFX is more scarce than for BNZ; unlike BZN, there is only one completed trial with 27 people treated and 24 placebo controls ^27^. According to a 2014 Cochrane systematic review, there is not robust evidence yet regarding efficacy on halting or delaying clinical progression ^28^. Before the BENEFIT (BENznidazole Evaluation For Interrupting Trypanosomiasis) trial (a randomized trial of BNZ for chronic Chagas’ cardiomyopathy) ^26^, observational studies had indicated a possible impact on disease progression and mortality. However, the BENEFIT trial was not able to demonstrate such effect ^29^. Criticisms about the design and very optimistic assumptions about the true effect of trypanocidal treatment have been raised about this trial. BENEFIT’s authors designed this trial assuming a high (26%) reduction relative to placebo in the incidence of cardiac complications among individuals with moderate to advanced cardiac disease. The trial identified a small (7%), but apparently consistent reduction of such outcomes, which may still be relevant for patients and from a public health perspective. For that small, but still relevant effect size, its sample size may have been underestimated (low-quality evidence for imprecision and lack of consistency with other studies, following GRADE criteria) ^30^. Using the current regimens of BNZ or NFX (60-day treatment course), only 70% of patients adhere to treatment on average, mostly due to adverse effects ^26^. However, alternative regimes with shorter duration or lower doses have been trialled (e.g. BENDITA trial ^31^) with promising results. This could lead to crucially improved adherence. Finally, an earlier diagnosis of cardiomyopathy and more comprehensive (supportive) treatment for heart failure may reduce mortality and hospitalizations by 20–30% (assuming, and yet to be tested, that such treatment has an effect similar to other causes of cardiovascular disease).

**Figure 3.**
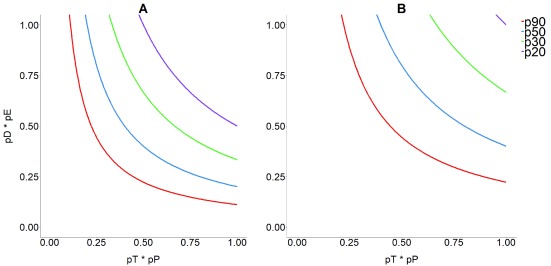
Probability of achieving effective parasite clearance. (A) 10% proportion of parasitological cure (PPC); and (B) 20% of PPC in a *Trypanosoma cruzi*–infected human population based on the combined probability of being diagnosed and treated (with trypanocidal medication) for Chagas disease. The horizontal axis represents the combined contribution of diagnosis as a product of the proportion of infected people who are tested (pT) and the proportion of those tested with a positive test result, that is, the sensitivity of the test (pP). The vertical axis represents the combined contribution of treatment, as the product of the proportion of those testing positives who are treated with currently available trypanocidal drugs (pD) and respond to treatment by clearing parasites according to efficacy (pE). Coloured lines represent the proportion (p) of infected people who would have to be reached by a test-and-treat programme (90% [blue], 50% [red], 33% [green], and 20% [orange]) to achieve the desired level of effective PPC. This figure has been reproduced from 17 under a Creative Common Attribution 4.0 International Licence (CC BY 4.0).

Modelling work has shown that an optimal combination of parameters such as: coverage of screening; performance of diagnostic tests; proportion of people treated; and efficacy of trypanocidal drugs is crucial to the scale-up of diagnosis and treatment programmes. While screening and access to treatment can be incremented as part of strengthening health systems, improving diagnostics performance and drug efficacy will require concerted efforts ^18^. With the current tools, low access to screening is the bottleneck; achieving just 10% of successful treatment at population level will require an enormous investment on improving access to screening, especially when targeting asymptomatic populations in low prevalence settings, which currently prevail in most endemic areas ^18^ ( Figure 3).

## Practical implications of the new targets

Achieving IDTI will require enhanced detection methods for domiciliated triatomines in low-infestation and low-prevalence settings. In order to monitor progress, improving the design of serological surveys for low-endemicity settings will be critical. FOI modelling suggests that increasing the age range for seromonitoring, instead of limiting it to under 5s or under 15s (e.g. using all-age classes), would be useful to understand temporal changes in *T. cruzi* incidence and the impact of interventions. Also, recent modelling studies have tested strategies to integrate data and models to guide interventions in Arequipa, Peru ^32^, which can be used to improve cost-effectiveness. Models of triatomine dispersal and colonization, with evidence-based parameterisation, are also needed to both understand their dynamics and design and test alternative control strategies ^22^.

Current estimates of access to diagnostics and treatment are at <1% ^33^. Reaching 75% by 2030 does not seem feasible using the current passive surveillance system. This goal would only be feasible if an optimized screening/treatment strategy is purposely designed for the targeted countries. Scaling-up current strategies needs a substantial commitment by such countries, and resource availability will be an issue. Also, the availability of current drugs is suboptimal, and there is a recurring need for supportive medical treatment of the CD sequelae.

In order to achieve 100% coverage of screening strategies implemented in blood banks and transplantation centres, a surveillance and administrative system needs to be put in place. Specifically, this requires more inter-sectorial collaboration, the participation of insurance companies, private/public institutions, the implementation of clear protocols, and a substantial commitment of health systems to ensure full documentation of the process. The WHO has a project in progress to develop an exhaustive database called the “World Information System for the Control of Chagas Disease” (WISCC), with an agreement with the Computer Centre of the Polytechnic University of Barcelona (Spain), that may be functional to these needs. Additionally, modelling could inform how widely screening should be done in non-endemic countries.

Achieving most of the goals currently stated for women and newborns seems challenging with the currently available tools ( Table 1). Given the long asymptomatic period and the current passive surveillance system for identifying cases of CD in endemic and non-endemic countries, it seems unfeasible that 90% of women of childbearing age will be screened. However, reaching almost 100% of pregnant women is potentially feasible with the recent strategy EMTCT plus (elimination of mother-to-child transmission of HIV, syphilis, Chagas, and perinatal hepatitis B), which adds mandatory surveillance tests during pregnancy for CD, planned to be in place in Colombia, Chile and Uruguay as pilot countries over the next few years ^34^. However, it is currently unrealistic to achieve 100% of treatment in newborns infected, as the sensitivity of micro-haematocrit methods (with repeated test) has been estimated at 34.2% ^35^. Repeated PCR-based tests can improve sensitivity up to 84.2%, but these tests are neither standardised nor widely available in endemic settings ^35^. Controlling congenital Chagas transmission would require urgent research for new diagnostics and drugs/drug regimes. Increased medical training and availability of tests and drugs would also need to be markedly improved. Higher sensitivity to detect congenital cases could also be achieved by including not only newborns, but also infants, for whom serological testing can be used from 8 months of age ^36^. Finally, careful planning and organization would also be essential for reaching and covering the more inaccessible rural (and indigenous) populations, which are likely to contribute disproportionately to the burden of CD.

## Risks and perverse incentives

Certifying intradomiciliary transmission interruption (IDTI) when such transmission has not truly been eliminated is the biggest risk. Current diagnostic tools are unlikely to be able to determine when true elimination has been achieved. However, it is perceived by public health officials that not having some reward system may harm even further the willingness of the countries towards elimination efforts. As countries reach low incidence, they may feel that efforts can be slackened. Determining the risk of re-colonisation after vector control is stopped and not having the tools to identify resurgence in a timely manner will, therefore, be important challenges. Developing and validating tools to quantify these risks will, in turn, inform ongoing initiatives to refine the process of re-certification that would follow after a (so far unspecified) number of years of the initial certification.

In terms of scaling-up treatment, it is anticipated that rare adverse effects of currently available drugs will become more evident. Also, in low-endemicity settings, the absolute number of false positives will be substantial as the number of people tested increase, even if specificity is high (98%, as estimated in a recent meta-analysis) ^37^. Similarly, treating false-positive cases can become a particularly important issue, as the absolute number of such cases will increase when diagnosis implementation and access increases.

## Moving towards elimination of transmission

Currently, endemic and non-endemic countries have been working on their own according to their priorities and resources, but a transition to realistic elimination goals at a global scale will require the concourse of both governmental and non-governmental organizations.

IDTI is only feasible in a few areas of exclusively domiciliated, non-native vectors. Once IDTI is achieved in a region, and the control programme is stopped, surveillance will be needed to detect resurgence. Currently, this occurs only over a few years after initial certification, but there is little knowledge and guidance available for this post-certification surveillance.

Large areas in endemic countries are populated by sylvatic triatomine species for which traditional vector control is not effective in a sustainable manner. For those areas more experimental and modelling work is needed to better understand both transmission and control strategies.

Scaling-up diagnosis and treatment strategies will require not only a greater commitment of the health systems but also an important investment in terms of R&D for diagnostics and treatment strategies.

## Modelling priorities to support goals in the 2030 horizon and beyond

Table 2 outlines the priority modelling questions for further research that were elaborated in discussion with the WHO.

**Table 2.** Summary of priority questions that can be answered with mathematical modelling.

Priority issue/question identified in discussions with WHO and PAHO	How can quantitative and mathematical modelling address this?
1. Monitoring progress towards 2030 goals	Generate projections of both transmission and disease burden at sub-national level.
2. Assess potential for resurgence when vector control interventions are stopped	Anticipate the risk and intensity of resurgence in different epidemiological scenarios and with different vector species.
3. Assess alternative control strategies for sylvatic vectors transmitting in both urban and rural areas	Understand sylvatic transmission cycles in both rural and urban areas and test alternative control strategies.
4. Evaluate the impact of scaling up diagnosis and treatment strategies	Predict the epidemiological impact of different strategies. Conduct cost-effectiveness analyses.

## Data (and software) availability

No data are associated with this article.
