# Supplementary material for: Insights from quantitative and mathematical modelling on the proposed WHO 2030 goals for Chagas disease
Source: Gates Open Res. 2019 Sep 17;3:1539. [Version 1] doi: 10.12688/gatesopenres.13069.1 (PMC6856696; doi:10.12688/gatesopenres.13069.1)
Supplement: Peer review history

# Peer review: Reviewer response for version 1

**DOI:** 10.21956/gatesopenres.14202.r27887

**Published:** 2019-10-24

**Authors:** Joel E. Cohen, Heinrich zu Dohna, Ricardo E Gürtler

**Affiliations:** 1 Laboratory of Populations, The Rockefeller University & Columbia University, New York, NY, USA; 2 American University of Beirut, Beirut, Lebanon; 3 IEGEBA-CONICET, Buenos Aires, Argentina

The Collaborating Group on Chagas Disease Modelling (CGCDM) gives a valuable review of the accomplishments and open challenges of quantitative and mathematical modelling of Chagas disease (CD). CGCDM provides a refreshingly frank critique of some goals of the World Health Organization (WHO), describing vector control in areas with sylvatic triatomine vectors as "not feasible in a sustainable manner" and the stated treatment goals as "not feasible" at all. The review provides convincing quantitative evidence for the latter assessment. It does not cite quantitative estimates to show that vector control in the face of sylvatic recolonization is unsustainable. The review would benefit from a more detailed quantitative description of what vector control efforts would be feasible in regions with multiple sources of recolonization, including sylvatic and between villages (e.g. Gürtler et al. 2007 ^1^).

 The assessment of CGCDM is likely to be useful to scientists who want to address critical gaps in modelling Chagas disease, to officers of WHO and national governments who seek to set realistic goals and make informed policy about CD control, and to funders in search of fact-based counsel on the opportunities and risks of supporting modelling research and field action to control CD.

 CGCDM strongly urges combining current vector control strategy with trypanocidal treatment of infected people. CGCDM identifies the enormous advances that will be required to achieve trypanocidal treatment of infected people. These advances include improved access to screening and diagnosis (currently <1% of individuals at risk have access in Colombia and perhaps elsewhere, according to previous sources and CGCDM), highly sensitive and highly specific tests to diagnose infection with *Trypanosoma cruzi*, more efficacious and less toxic drugs or other treatments than are currently available, and persuading people to complete the course of treatment. CGCDM also candidly identifies some of the risks of its own recommended strategy.

 Based on experience in CD vector and disease control in the semiarid and humid Chaco of northern Argentina, currently the main hotspot of vector-borne transmission, we suggest three additional ingredients that may enhance the recipes advanced by CGCDM for interrupting vector-borne transmission of *T. cruzi* to people. We believe that these ingredients would prove valuable to CD control in regions of vector-borne transmission beyond the Argentine Chaco, but that remains to be proved. These ingredients are: (1) engaging the families and communities affected by CD in protecting themselves from CD, and modelling their engagement; (2) enriching the picture of CD dispersal using genetic and other modern techniques, and modelling that more complex picture; and (3) improving the rationality and statistical efficiency of vector control, and modelling the resulting impacts on parasite transmission and economic savings from improved vector detection and control. Our suggestions do not address other important problems CGCDM addressed, such as interrupting transmission of *T. cruzi* by blood transfusion and transplants, increasing access to expanded diagnostic and treatment programs, and preventing and controlling congenital transmission. We now sketch each proposed additional ingredient.

 (1) Engaging the communities and families affected by CD in protecting themselves

 CGCDM do not mention the roles of the individuals, families, and communities affected by CD in defending themselves. We think these roles could be influential, perhaps even crucial, in controlling and monitoring CD, and achieving the 2030 targets identified in Table 1. Specifically, people living in areas where vector-borne CD is endemic can influence wall and roof construction and maintenance, domestic animal management, on-demand residual insecticide spraying, and monitoring any resurgence of bugs after official interventions (e.g., Monroy et al. 2009 ^2^). Community participation and training are key to achieving a highly demanding goal such as “0% colonization rate of dwellings” in the affected zones.

 In the Gran Chaco region, a single village is typically composed of multiple house compounds. Each house compound consists of one (or more) domiciles (for human residents) and multiple outbuildings, such as chicken coops, goat corrals, kitchens, grain stores, and outhouses. Domiciles of different house compounds, though constructed with the same basic materials (mud bricks and poles), vary widely in the state of repair of the walls. Walls smoothly plastered provide no refuge for the triatomine vectors of CD and the human residents typically suffer lower incidence of *T. cruzi*. By contrast, houses with cracked walls shelter the bugs, have higher triatomine infestation levels (Cecere et al. 2002 ^3^; Bustamante et al. 2009 ^4^), and the human residents are more likely to be infected with *T. cruzi* across settings and vector species (e.g., Mott et al. 1978 ^5^, Andrade et al. 1995 ^6^, Bonfante-Cabarcas et al. 2011 ^7^). Similarly, house compounds that have thatched roofs provide good refuges for bugs. Domiciles roofed with materials less hospitable to resting bugs usually have lower prevalence and incidence of *T. cruzi*. (Gürtler et al. 1998 ^8^; Black et al. 2007 ^9^). Domestic dogs have a very high prevalence of infection with *T. cruzi* and a very high probability of transmitting *T. cruzi* to uninfected bugs. When bugs thus infected are in human sleeping quarters, they have an increased likelihood of transmitting *T. cruzi* to humans. Keeping domestic dogs out of human sleeping quarters permanently could substantially reduce the incidence of human infection, according to modelling (Cohen and Gürtler 2001 ^10^) and observations in Argentina (Gürtler et al. 2005 ^11^, Cardinal et al. 2018 ^12^). In Venezuela, the household presence of several domestic animal species (dogs, caprines, armadillos and chickens) is significantly and positively associated with human seroprevalence (Bonfante-Cabarcas et al. 2011 ^7^).

 Because of the long asymptomatic period of CD, residents in endemic areas often have a low awareness of the risks associated with *Triatoma infestans* infestation. Local residents could be educated about the risks of bug-mediated transmission of *T. cruzi* to their own health and the health of their children. They could also be taught how to apply residual insecticides (individually purchased or provided by control agencies) as need arises within their domiciles and outbuildings, and to monitor the resurgence of bugs after control measures (Cecere et al. 2019 ^13^, Gaspe et al. 2018 ^14^). Thatch-roofed granaries are not usually monitored or sprayed, may often harbor large bug populations, and could be a potent source of bug reinfestation of other structures (Cohen et al. 2017 ^15^).

 Modelling challenges are to synthesize what is known about the magnitudes of these effects and to compare the costs and benefits of house-to-house and community-wide education about walls, roofs, domestic dogs, spraying, and monitoring with the costs and benefits of more traditional top-down interventions. The latter have proven difficult to sustain in the long run, especially when intrusive triatomine species tend to invade domestic premises and pose a recurrent risk of recolonisation, such as with *Triatoma dimidiata* in Central America (Peterson et al. 2019 ^16^) and *Rhodnius prolixus* in Colombia and Venezuela (Sanchez-Martin et al. 2006 ^17^, ref. 12).

 (2) Enriching the picture of CD vector dispersal

 Based on modelling studies, CGCDM "suggest that a better comprehension of vectorial transmission in rural and urban settings would require understanding and quantifying of two different forms of vector dispersal, namely, dispersal between sylvatic and non-sylvatic habitats and diffusive dispersal within cities." We agree with the need to understand both forms of dispersal. We propose that additional levels of dispersal need empirical studies and modelling.

 Within a house compound, bugs disperse among domiciliary and peri-domiciliary structures. Within a village, bugs disperse among house compounds. Within localities or clusters of villages, bugs disperse among villages possibly through flight and possibly through movements of people, non-human animals, and goods. At all three levels (house compound, village, cluster of villages), bugs may disperse between sylvatic and settled habitats, and all forms of bug dispersal may depend on the season (Dohna et al. 2007 ^18^, 2009 ^19^; Gourbière et al. 2008 ^20^). Independently of the dispersal of bugs, people (and their dogs and cats) carry infections of *T. cruzi* among house compounds, villages, localities, urban and rural regions of a given country, and internationally. Four multi-level networks of dispersal interact: bugs, humans, domestic animals, and *T. cruzi* (in bugs, people, and domestic animals).

 In future empirical work, genetic techniques to infer the origins and spread of infections (Biek et al. 2012 ^21^; Gourbière et al. 2012 ^22^; Gire et al. 2014 ^23^; Grad et al. 2014 ^24^) need to be tailored and applied to the origins and transmission of *T. cruzi* infections and the dispersal of the vectors. For example, Nouvellet et al. (2013) ^25^ used the incidence of *T. cruzi* infections in humans and the average number of potentially infective contacts per uninfected person to estimate that the probability of *T. cruzi* transmission per potentially infective contact is 1 in 900-4000 contacts with infected bugs. If genetic techniques were used to identify markers or "barcodes" in *T. cruzi*, the appearance of these markers in people newly infected with *T. cruzi* could be used to confirm or refine these estimates.

 Modelling challenges are to model the structures and infer the parameter values of these networks of interactions using available data (e.g., Dohna and Pineda-Krch 2010 ^26^) and to design additional practical data collection goals for improved modelling and estimation.

 (3) Improving the rationality and statistical efficiency of vector control by spraying insecticides

 A decade ago, Dohna et al. (2009 ^19^, p. 1) made a statement that was true then and unfortunately remains true today: "… it is important to increase the efficiency of vector control. It is currently unknown what spatial and temporal pattern of repeated insecticide application maximizes its efficiency." Some modeling studies (reviewed in Nouvellet et al. 2015 ^27^) produced concrete recommendations for optimizing vector control but none of these recommendations has been tested in field trials. Large-scale field trials that test well-founded model-based recommendations for cost-effective vector control are crucial, especially for areas where resources are limited and sustained vector control is required.

 Mass spraying of residual insecticides in a community is traditionally triggered when the community-wide fraction of bug-infested houses (the house infestation rate) detected by a standardized search procedure exceeds a fixed threshold, typically 5% (Schofield 1994 ^28^; Guhl 2005 ^29^, p. 395; Aiga et al. 2012 ^30^ suggest a threshold of 8% for their region). The number of houses to be searched is rarely defined in advance, or all houses in a village are searched, which is costly and time-consuming.

 A more efficient alternative could be to design a plan of sequential sampling that stops searching of houses and starts spraying when the probability that the infestation rate exceeds 5% reaches a chosen standard. For example, suppose the probability of house infestation is 5% or less. If houses are chosen randomly from a list of houses in the village, then the probability that the first three houses are all infested is at most (0.05) ^3^ = 0.000125, just over one chance in ten thousand. In this case, it would seem superfluous to search additional houses for bug infestation before deciding to spray the entire community. The specification in this example that "houses are chosen randomly from a list of houses in the village" is important, because if the inspectors choose geographically contiguous houses for their own convenience rather than randomly, there may be dependence in the infestation of successive neighboring houses. For example, three successive houses might be infested as a result of their proximity or shared familial indifference to plastering walls, regardless of the house infestation rate of the community. Sequential plans for detecting pest infestation rates above an economic threshold are commonly used in commercial agriculture. Similar procedures need to be developed and adapted for the efficient surveying of house infestations in the control of CD. Sampling design is of prime relevance, especially when control programs pursue certification of transmission interruption or vector elimination.

 Modelling challenges include designing sequential sampling plans that take account of the consistent relationships between the mean and the variance of bug relative population sizes in different habitats within house compounds (Taylor's law; Cohen et al. 2017 ^15^); modelling and measuring the spatial aggregation of house infestation; and estimating the economic savings resulting from more efficient vector detection and control.

 The paper ably draws attention to some undesirable consequences of the certification of IDTI witnessed during the post-certification period in several countries. In general, CD vector control programs have lost substantial operational capacity. The (re)emergence and growing relevance of dengue and of other mosquito-borne viruses such as zika and chikungunya have drawn away from CD the scarce resources available for vector control. This complex scenario in part explains why ten countries have yet to hit the goal of IDTI by 2020 (Tarleton et al. 2014 ^31^). Competition for scarce resources for vector control remains a major obstacle to hitting the 2030 targets.

 The following passages contain some inaccuracies or imprecise language and should be reviewed.

 In the Abstract, "Modelling insights have shown that IDTI is feasible in areas with sustained vector control programmes and no presence of native triatomine vector populations." An interested reader not informed of the main issue under discussion may not understand the underlying logic of this sentence. If there is no presence of native triatomine populations, neither IDTI nor sustained triatomine control programmes would be pertinent, unless there are other (non-native) vectors that pose a risk of human infection with *T. cruzi*.

 The Abstract begins: "Chagas disease (CD) persists as one of the neglected tropical diseases (NTDs) with a particularly large impact in the Americas" and the Background section on page 3 continues: "CD is a parasitic disease caused by the protozoan *Trypanosoma cruzi*, and transmitted mainly by domiciliated triatomine (Reduviidae) vectors (kissing bugs) in tropical areas of the Americas." In fact, the trypanosome that causes CD is transmitted mainly by domiciliated triatomine (Reduviidae) vectors (kissing bugs) in tropical, subtropical and temperate areas of the Americas. CD is not restricted to tropical areas.

 The Background section, page 3, states: “…along with the urbanization process in recent decades, other transmission routes, such as blood transfusion, organ transplant and congenital have become important in both endemic and non-endemic countries ^2^.” This generalization is partially inaccurate. In ref. 2, Table 2 shows no transfusion-related CD case in 21 Latin American endemic countries. This is quite plausible because screening of blood donors has achieved virtually universal coverage decades ago, with rare exceptions. A 2015 WHO publication, based on 2010 data provided by the countries, reported that “19 out of the 21 endemic countries achieved 100% screening of donated blood.” The issue of transfusion-mediated transmission has been or still is pertinent to some non-endemic, developed countries.

 The Background section, page 3, also states: "So far, 11 out of the 21 recognised endemic countries have been certified as having reached intradomiciliary transmission interruption (IDTI)." This statement inaccurately suggests that there is no vector-borne domestic transmission of *T. cruzi* in 11 countries. The certifications of IDTI for Central American countries are relevant for *R. prolixus*, not for *T. dimidiata*, a widespread vector involved in domestic transmission to humans (Peterson et al. 2019 ^16^). Similarly, the certifications for the Southern Cone countries are relevant for *T. infestans*, but in Brazil there are other species involved in domestic transmission.

 On page 4, a reference is needed to justify: "the number of triatomines/house was fitted to data from various locations prior to vector control and applied to cases where there was only one triatomine species present in the dwellings, as well as to situations with mixtures of species and developmental stages, various types of houses and bug densities per house."

 Also on page 4: "… In the presence of sylvatic populations, there is a continuous introduction and colonisation of domiciliary and peri-domiciliary habitats; in these areas, traditional vector control is not feasible in a sustainable manner." A better distinction between feasibility, effectiveness and sustainability is relevant. Many decades ago, in some of these areas, such as in Venezuela, traditional vector control was feasible and very effective by current standards when it was pursued consistently (Feliciangeli et al. 2003 ^32^, Bonfante-Cabarcas et al. 2011 ^7^, Bartsch et al. 2017 ^33^). The decentralization of healthcare services, beginning in the late 1980-1990s in Latin America, combined with other regional processes, virtually eliminated or downsized the existing vector control programs except in a few countries.

 Also on page 4: "Various studies on routine vector surveillance have demonstrated that the currently used methods have low sensitivity and greatly underestimate vector density, infestation and infection rates; vector surveillance may be capturing half of infestations – and, most likely, most bugs within a house ^13,14^." …"most bugs within a house" suggests that the methods may be capturing most of the bugs within the house, which disagrees with the former sentence and field observations. Perhaps the sentence intended to say that vector surveillance was detecting half of infestations, and standard vector collection methods captured mostly bugs within a house, i.e., domiciles.

 On page 6: "Trypanocidal treatment with benznidazole (BNZ) or nifurtimox (NFX) has been aimed at both reducing parasitaemia and curbing disease progression. So far, there is limited evidence on the efficacy of drugs for these." There is substantial evidence on the efficacy of BNZ in suppressing or reducing parasitaemia as determined by RT-PCR (e.g., ref. 24, 28), whereas the available evidence on curbing disease progression is limited and debatable (e.g., ref. 29). The age of the patient under treatment is important: both drugs are able to achieve the serological and parasitological cure of recent chronic infections when applied to *T. cruzi*-seropositive children under age 15 years (ref. 28), whereas in chronic adult patients both drugs suppressed or reduced parasitaemia albeit transiently, as determined by PCR or xenodiagnosis (ref. 24, 28); the serological effects of treatment with either drug may take many years to appear.

 On page 7: "With the current tools, low access to screening is the bottleneck; achieving just 10% of successful treatment at population level will require an enormous investment on improving access to screening, especially when targeting asymptomatic populations in low prevalence settings, which currently prevail in most endemic areas ^18^". This issue is debatable and likely varies geographically. In Argentina, the key obstacle has been access to treatment in rural areas: either the drugs or the treating physicians are not available to indicate treatment and manage the adverse drug-related reactions over the 60-day follow-up (Sartor et al. 2017 ^34^). Health services and several NGOs (e.g. MSF) have conducted large serosurveys of *T. cruzi* infection in rural areas using rapid tests or dried blood spots.

 Also on page 7: "Current estimates of access to diagnostics and treatment are at <1% ^33^." The pertinent reference is Ribeiro et al. (2009) ^35^, who provided the <1% estimate across endemic countries. Ref. 33 reported that access to diagnostics and treatment in Colombia was 1.2%.

 A few editorial errors should be corrected. In the caption of Figure 3, the identification of the coloured lines is wrong. The colour code adjacent to the upper right corner of panel B is more likely to be correct. In line 5 of paragraph 2 on page 6, BZN should be replaced by BNZ. In the captions of Fig. 2 and 3, ref. 17 should be replaced by ref. 18. Table 1 should list the 21 endemic countries, in line with recommendations issued by PAHO/WHO (2018) ^36^ and other passages in the text.

We confirm that we have read this submission and believe that we have an appropriate level of expertise to confirm that it is of an acceptable scientific standard.

## References

[1] : Sustainable vector control and management of Chagas disease in the Gran Chaco, Argentina. *Proc Natl Acad Sci U S A*.2007;104(41) : 10.1073/pnas.0700863104 16194-9 10.1073/pnas.0700863104 17913895PMC2042184

[2] : House improvements and community participation in the control of Triatoma dimidiata re-infestation in Jutiapa, Guatemala. *Cad Saude Publica*.2009;25 Suppl 1: 10.1590/s0102-311x2009001300016 S168-78 10.1590/s0102-311x2009001300016 19287861

[3] : Effects of partial housing improvement and insecticide spraying on the reinfestation dynamics of Triatoma infestans in rural northwestern Argentina. *Acta Tropica*.2002;84(2) : 10.1016/S0001-706X(02)00183-3 101-116 10.1016/S0001-706X(02)00183-3 12429427

[4] : Risk factors for intradomiciliary infestation by the Chagas disease vector Triatoma dimidiatain Jutiapa, Guatemala. *Cad Saude Publica*.2009;25 Suppl 1: 10.1590/s0102-311x2009001300008 S83-92 10.1590/s0102-311x2009001300008 19287870

[5] : House construction, triatomine distribution, and household distribution of seroreactivity to Trypanosoma cruzi in a rural community in northeast Brazil. *Am J Trop Med Hyg*.1978;27(6) : 10.4269/ajtmh.1978.27.1116 1116-22 10.4269/ajtmh.1978.27.1116 103445

[6] : Evaluation of risk factors for house infestation by Triatoma infestans in Brazil. *Am J Trop Med Hyg*.1995;53(5) : 10.4269/ajtmh.1995.53.443 443-7 10.4269/ajtmh.1995.53.443 7485701

[7] : [Seroprevalence for Trypanosoma cruzi infection and associated factors in an endemic area of Venezuela]. *Cad Saude Publica*.2011;27(10) : 10.1590/s0102-311x2011001000005 1917-29 10.1590/s0102-311x2011001000005 22031196

[8] : Household prevalence of seropositivity for Trypanosoma cruzi in three rural villages in northwest Argentina: environmental, demographic, and entomologic associations. *Am J Trop Med Hyg*.1998;59(5) : 10.4269/ajtmh.1998.59.741 741-9 10.4269/ajtmh.1998.59.741 9840591

[9] : Household risk factors for Trypanosoma cruzi seropositivity in two geographic regions of Ecuador. *J Parasitol*.2007;93(1) : 10.1645/GE-899R.1 12-6 10.1645/GE-899R.1 17436937

[10] : Modeling household transmission of American trypanosomiasis. *Science*.2001;293(5530) : 10.1126/science.1060638 694-8 10.1126/science.1060638 11474111

[11] : Incidence of trypanosoma cruzi infection among children following domestic reinfestation after insecticide spraying in rural northwestern Argentina. *Am J Trop Med Hyg*.2005;73(1) :95-103 16014842PMC1351233

[12] : High levels of human infection with Trypanosoma cruzi associated with the domestic density of infected vectors and hosts in a rural area of northeastern Argentina. *Parasit Vectors*.2018;11(1) : 10.1186/s13071-018-3069-0 492 10.1186/s13071-018-3069-0 30165892PMC6118006

[13] : Community-based surveillance and control of chagas disease vectors in remote rural areas of the Argentine Chaco: A five-year follow-up. *Acta Trop*.2019;191: 10.1016/j.actatropica.2018.12.038 108-115 10.1016/j.actatropica.2018.12.038 30593817

[14] : Beating the odds: Sustained Chagas disease vector control in remote indigenous communities of the Argentine Chaco over a seven-year period. *PLoS Negl Trop Dis*.12(10) : 10.1371/journal.pntd.0006804 e0006804 10.1371/journal.pntd.0006804 30278044PMC6168123

[15] : Chagas disease vector control and Taylor's law. *PLoS Negl Trop Dis*.2017;11(11) : 10.1371/journal.pntd.0006092 e0006092 10.1371/journal.pntd.0006092 29190728PMC5734788

[16] : Chagas Disease in Central America: Recent Findings and Current Challenges in Vector Ecology and Control. *Current Tropical Medicine Reports*.2019;6(2) : 10.1007/s40475-019-00175-0 76-91 10.1007/s40475-019-00175-0

[17] : Could the Chagas disease elimination programme in Venezuela be compromised by reinvasion of houses by sylvatic Rhodnius prolixus bug populations?. *Trop Med Int Health*.2006;11(10) : 10.1111/j.1365-3156.2006.01717.x 1585-93 10.1111/j.1365-3156.2006.01717.x 17002733

[18] : Re-establishment of local populations of vectors of Chagas disease after insecticide spraying. *J Appl Ecol*.2007;44(1) : 10.1111/j.1365-2664.2006.01243.x 220-227 10.1111/j.1365-2664.2006.01243.x 17710182PMC1948873

[19] : Spatial re-establishment dynamics of local populations of vectors of Chagas disease. *PLoS Negl Trop Dis*.2009;3(7) : 10.1371/journal.pntd.0000490 e490 10.1371/journal.pntd.0000490 19636363PMC2709728

[20] : Demographic and dispersal constraints for domestic infestation by non-domicilated chagas disease vectors in the Yucatan Peninsula, Mexico. *Am J Trop Med Hyg*.2008;78(1) :133-9 18187796

[21] : Whole genome sequencing reveals local transmission patterns of Mycobacterium bovis in sympatric cattle and badger populations. *PLoS Pathog*.2012;8(11) : 10.1371/journal.ppat.1003008 e1003008 10.1371/journal.ppat.1003008 23209404PMC3510252

[22] : Genetics and evolution of triatomines: from phylogeny to vector control. *Heredity (Edinb)*.2012;108(3) : 10.1038/hdy.2011.71 190-202 10.1038/hdy.2011.71 21897436PMC3282395

[23] : Genomic surveillance elucidates Ebola virus origin and transmission during the 2014 outbreak. *Science*.2014;345(6202) : 10.1126/science.1259657 1369-72 10.1126/science.1259657 25214632PMC4431643

[24] : Improving Control of Antibiotic-Resistant Gonorrhea by Integrating Research Agendas Across Disciplines: Key Questions Arising From Mathematical Modeling. *J Infect Dis*.2016;213(6) : 10.1093/infdis/jiv517 883-90 10.1093/infdis/jiv517 26518045PMC4760416

[25] : The improbable transmission of Trypanosoma cruzi to human: the missing link in the dynamics and control of Chagas disease. *PLoS Negl Trop Dis*.2013;7(11) : 10.1371/journal.pntd.0002505 e2505 10.1371/journal.pntd.0002505 24244766PMC3820721

[26] : Fitting parameters of stochastic birth-death models to metapopulation data. *Theor Popul Biol*.2010;78(2) : 10.1016/j.tpb.2010.06.004 71-6 10.1016/j.tpb.2010.06.004 20600207

[27] : Ecology, evolution and control of Chagas disease: a century of neglected modelling and a promising future. *Adv Parasitol*.2015;87: 10.1016/bs.apar.2014.12.004 135-91 10.1016/bs.apar.2014.12.004 25765195

[28] : Triatominae biology and control. *Transactions of the Royal Society of Tropical Medicine and Hygiene*.1994;88(5) : 10.1016/0035-9203(94)90187-2 10.1016/0035-9203(94)90187-2

[29] : Chagas disease: assessing the existence of a threshold for bug infestation rate. *Am J Trop Med Hyg*.2012;86(6) : 10.4269/ajtmh.2012.11-0652 972-9 10.4269/ajtmh.2012.11-0652 22665603PMC3366542

[30] : Chagas disease and the london declaration on neglected tropical diseases. *PLoS Negl Trop Dis*.2014;8(10) : 10.1371/journal.pntd.0003219 e3219 10.1371/journal.pntd.0003219 25299701PMC4191937

[31] : Chagas disease control in Venezuela: lessons for the Andean region and beyond. *Trends Parasitol*.2003;19(1) :44-9 1248822610.1016/s1471-4922(02)00013-2

[32] : Comparison and validation of two computational models of Chagas disease: A thirty year perspective from Venezuela. *Epidemics*.18: 10.1016/j.epidem.2017.02.004 81-91 10.1016/j.epidem.2017.02.004 28279459PMC5549789

[33] : Improving access to Chagas disease diagnosis and etiologic treatment in remote rural communities of the Argentine Chaco through strengthened primary health care and broad social participation. *PLoS Negl Trop Dis*.11(2) : 10.1371/journal.pntd.0005336 e0005336 10.1371/journal.pntd.0005336 28192425PMC5325580

[34] : New, improved treatments for Chagas disease: from the R&D pipeline to the patients. *PLoS Negl Trop Dis*.2009;3(7) : 10.1371/journal.pntd.0000484 e484 10.1371/journal.pntd.0000484 19582163PMC2702098

[35] Chagas Disease in the Americas: A Review of the Current Public Health Situation and a Vision for the Future. *PAHO and WHO*.2018;

[36] Memorias del Primer Taller Internacional sobre Control de la Enfermedad de Chagas. *Universidad de los Andes*.2005; Reference source

# Peer review: Reviewer response for version 1

**DOI:** 10.21956/gatesopenres.14202.r27971

**Published:** 2019-10-17

**Authors:** María del Carmen Fabrizio, Nicolás J. Schweigmann

**Affiliations:** 1 Department of Quantitative Methods and Information Systems, Faculty of Agronomy, University of Buenos Aires, Buenos Aires, Argentina; 2 Department of Ecology, Genetics and Evolution, Faculty of Exact and Natural Sciences, University of Buenos Aires, Beunos Aires, Argentina

Authors show the current situation state of Chagas infection and how mathematical modelling can help in carrying out the WHO goals proposed for 2030. The infection by *Trypanosoma cruzi* (causal parasite) is mainly transmitted through haematophagous triatomine insects, as a consequence of migration waves has considerably expanded from rural endemic areas to urban centers. This idea is mentioned in the first paragraph of Background section. (“ However along with the urbanization process…have become important in both endemic and non-endemic countries”). We suggest presenting current numbers in Belgium, Italy, United Kingdom, Switzerland, Spain, Australia, Japan, among others because this is an unknown infection. Moreover, most of the physicians do not know the illness ethiology. (Freilij *et al.*, 2007 ^1^; Lugo Caballero *et al.*, 2017 ^2^).

 Authors are critical according to the knowledge about  “11 out of 21 recognized endemic countries have been certificated as having reached intradomiciliary transmission interruption” (Second paragraph of Background section). We agree that multiple actors are involved in vectorial transmission in rural zones of America: sylvan triatomines, which mediate between sylvan and peridomestic cycles. Dogs are domestic animals living with humans, which spread the infection although they do not transmit the infection to humans and other animal species as food sources for triatomines (e.g. poultry).     

 We also agree with “However, the real impact of such interventions has not been rigorously documented and quantified, and various concerns have been raised around the relationship between reaching the various operational thresholds that have been proposed and truly achieving interruption/elimination of transmission and reduction in morbidity. Also, the increased health-care demands from the chronically-affected population, and the limited offer of diagnosis and trypanocidal and supportive treatment pose additional challenges to Chagas disease control”.

 Authors suggest the necessity to count with more studies for understanding the relationships among some critical co-variates, such as house construction materials, number of humans and zoonotic hotos” and “even if the domiciliated vector species are eliminated, their niche could be taken over by other sylvan vector species”.

 We also want to remind that other transmission route from *T, cruzi* to human beings in América is oral transmission, which was not mentioned by authors. In recent years a significant increase in the oral transmission was observed associated with the consumption of drinks made from fruit or other vegetables contaminated with triatomines faeces or secretions from infected mammals or by the ingestion of undercooked meat from infested host animals. Mortality reachs up 33% of those infected people (Filigheddu *el al*., 2017 ^3^). Environment changes such as deforestation and global warming have affect ecotopes and the behaviour of *T. cruzi* vectors and reservoirs so that these have become displaced to new areas, thereby heading to such transmission scenario caused by contamination food , which requires evaluation (Rueda *el al*., 2014 ^4^).

 Other mentioned problem is about the diagnostic tests do not have perfect sensitivity and specificity.

 “Another main target planned is achieving 75% access to trypanocidal treatment in *T. cruzi* infected people”, when “current estimates of access to diagnostics and treatment are at <1%”. And another problem is that there is limited evidence on the efficacy of drugs used currently.

 The Letter is indexable and contributes considerably to show the infection situation by *T.cruzi* , in  rural areas from Latin America, America, Europe and Western Pacific. The article proposes the important discussion of control measures, searching probable infected people and the corresponding treatment.

 Mathematical models are an effective tool for organizing ideas and making predictions and future planifications. These models permit to observe the consequences of the chosen premises. Otherwise, the usefulness model is the accuracy of the parameters estimates. This goal may be reach with the interdisciplinary work involving biologists, ecologists, architects, physicians from different areas (obstetricians, paediatricians, cardiologists, neumonologists), epidemiologists and mathematicians.

 The 2020 is very near to begin, the year when a current WHO goal is Interruption of intradomiciliary (vectorial) transmission in the Americas. We believe good mathematical models are necessary and as the authors expressed,“the concourse of both governmental and non-governmental organizations” .It is very necessary to get tools for the evaluation of goals compliance from every country/region/province according to their sociopolitical and economical situations because of there are several federal countries, which are depending on every province decisions. Socio politics and economic crisis in South America complicate the monitoring over the states of vectorial situation, carrying out sustained politics for enhancing humans life quality.

 Besides, the lack of control in blood banks or organ transplants and pregnant women has caused the spread of the disease in American, European, Australian and Japan cities (Kirchhoff 2011 ^5^, WHO 2011 ^6^). Outside of rare specialized center, health care provides lack of experience, diagnosing and treating the disease, with subsequent disease progress and dissemination of the infection (Traina *et al.* 2016 ^7^). And recent development finding in many countries, especially in United States of America, Asia and Australia, of Chagas disease vectors capable of transmitting the infection and thereby raising the risk of establishing vector transmission in these countries (WHO, 2015 ^8^).

We confirm that we have read this submission and believe that we have an appropriate level of expertise to confirm that it is of an acceptable scientific standard.

## References

[1] : [Chagas disease in the Americas: an ecohealth perspective]. *Cad Saude Publica*.2009;25 Suppl 1: 10.1590/s0102-311x2009001300007 S71-82 10.1590/s0102-311x2009001300007 19287869

[2] : [Knowledge of vector-borne diseases (dengue, rickettsiosis and Chagas disease) in physicians]. *Gac Med Mex*.153(3) :321-328 28763070

[3] : Orally-transmitted Chagas disease. *Med Clin (Barc)*.2017;148(3) : 10.1016/j.medcli.2016.10.038 125-131 10.1016/j.medcli.2016.10.038 27993415

[4] : [Oral transmission of Trypanosoma cruzi : a new epidemiological scenario for Chagas' disease in Colombia and other South American countries]. *Biomedica*.34(4) : 10.1590/S0120-41572014000400017 631-41 10.1590/S0120-41572014000400017 25504253

[5] : Epidemiology of American trypanosomiasis (Chagas disease). *Adv Parasitol*.2011;75: 10.1016/B978-0-12-385863-4.00001-0 1-18 10.1016/B978-0-12-385863-4.00001-0 21820549

[6] : Informal Consultation on Chagas Disease in the Western Pacific. *Meeting Report*.2011; Reference source

[7] : Heart Failure Secondary to Chagas Disease: an Emerging Problem in Non-endemic Areas. *Curr Heart Fail Rep*.13(6) : 10.1007/s11897-016-0305-9 295-301 10.1007/s11897-016-0305-9 27807757

[8] Investing to Overcome the Global Impact of Neglected Tropical Diseases. *Third WHO Report of Neglected Tropical Diseases*.2015; Reference source
